# Treatment of Stomatitis Materna by the Syrup of the Phosphates

**Published:** 1860-01

**Authors:** E. J. Fountain

**Affiliations:** Davenport, Iowa


ART. III.—*Treatment of Stomatitis Materna by the Syrup of the Phosphates.* By E. J. FOUNTAIN, M.D., of Davenport, Iowa.

IN the last number of this journal is reported, by Dr. J. C. Reeves, of Ohio, a case of stomatitis materna, successfully treated by the syrup of the phosphates. In conclusion, the writer remarks: "It is a single case, and as such is only valuable as encouraging to those who have cases upon which they have exhausted remedies and patience, to make one more trial."

This invitation to the profession induces me to communicate the result of my experience in the same treatment of this disease.

Where the encouragement of hæmatosis is so plainly indicated as in stomatitis materna, it is not surprising that the so-called "chemical food" should have been suggested as a suitable remedy to a number of practitioners, independently of each other. All, however, must be indebted very much to Dr. David Hutchinson, of Indiana, whose excellent exposition of the pathology of this disease, in the "Fiske Fund Prize Essay" of 1857, plainly suggests the use of blood-making remedies.

At the last meeting of the Iowa State Medical Society, held at Davenport, June, 1859, I verbally reported the successful treatment of several cases of stomatitis materna by the use of the syrup of the phosphates.

Upon the appointment of a committee to report on this disease at our next meeting, I called the attention of the Society to the use of this remedy, which I had tried in four well-marked cases with happy effect, and recommended the members to give it a trial in their practice, to test its value.

It is now generally conceded that this disease is the result of a peculiar impoverishment of the blood, resulting from the tax upon its substance by the development of the fœtus, and subsequent lactation. Naturally, the functions of digestion and assimilation are adequate to the supply of this demand, but the tendency of modern civilization and fashion leads to such a derangement of the digestive organs that we often find ladies assuming the responsible duties of maternity who have scarcely vitality sufficient to support their own feeble constitutions. The wonder is, not that nursing sore-mouth is so frequent in our day, but that kind nature does not more frequently protest against the ill-treatment imposed upon her.

Sympathy with the gravid uterus undoubtedly increases the debility of the digestive organs when a predisposition exists to such a difficulty; but without this predisposition I believe it will not, of itself, produce it. The indication is, therefore—first, to improve the tone of the digestive apparatus; and next, to supply it with such nutriment as will be most readily assimilated into good blood, and especially such as is needed for fœtal growth and subsequent lactation. When the tax upon the blood has been greater than the supply, it is proper to furnish something which will quickly restore the balance, by which the excess of fibrin will be reduced, and its peculiar effect (the precise connection with which is not yet well understood) upon the mucous membranes of the mouth and alimentary canal be arrested, and the general health restored. By this view of its pathology the plan of treatment is clearly indicated; and the syrup of the phosphates, or "chemical food," occurred to me as an appropriate remedy. On its first trial the disease soon disappeared, but I thought the recovery might be due to natural causes, uninfluenced by the treatment. After this, I had an opportunity of testing it in three cases, in each of which the administration of the remedy was soon followed by complete recovery. In one very severe case I had to aid the treatment by adjuvants, to meet particular indications: as opiates and astringents to check a diarrhœa, and bismuth and antacids to correct disordered digestion; and the recovery was further promoted by the use of cod-liver oil, as recommended by Drs. Evans and Byford, of Chicago.

These cases impressed my mind with the belief that, in the syrup of the phosphates, freely administered, we had a pleasant and efficient remedy for the fulfillment of the most important indication. In simple and mild cases it has appeared to be the only remedy needed; but in those of a more aggravated character it cannot be depended upon as a specific, but must be aided by other treatment appropriate to particular symptoms.

In the case last referred to, I increased the strength of the syrup by the addition of an extra quantity of the phosphate of iron and phosphate of lime, giving to the patient five grains of each with every dose, and finally administered in connection with cod-liver oil.

The cases here mentioned, together with the one reported by Dr. Reeves, are not sufficient to establish the remedy as one that can always be depended upon; but they furnish a strong presumption in favor of the treatment, and in confirmation of the theory by which it was suggested.

